# Outcome and long-term efficacy of four facio-cervical fields conformal radiotherapy for nasopharyngeal carcinoma

**DOI:** 10.18632/oncotarget.14403

**Published:** 2016-12-31

**Authors:** Wang Fangzheng, Jiang Chuner, Wang Lei, Chen Weijun, Xu Min, Sun Quanquan, Liu Tongxin, Rihito Aizawa, Masoto Sakamoto, Fu Zhenfu

**Affiliations:** ^1^ Department of Radiation Oncology, Zhejiang Cancer Hospital, Zhejiang Hangzhou, 310022 China; ^2^ Zhejiang Key Laboratory of Radiation Oncology, Zhejiang Hangzhou, 310022 China; ^3^ Department of Breast Surgery, Zhejiang Cancer Hospital, Zhejiang Hangzhou, 310022 China; ^4^ Department of Radiology, Japanese Red Cross Fukui Hospital, Fukui, 918-8501 Japan

**Keywords:** nasopharyngeal neoplasm, conformal radiotherapy, chemotherapy, efficacy, prognosis

## Abstract

**Purpose:**

To evaluate the outcomes of 255 patients with nasopharyngeal carcinoma (NPC) treated with four facio-cervical fields conformal radiotherapy (4F-CRT).

**Results:**

In one patient's 3 different RT treatment modalities, the 4F-CRT techniques resulted in sharper of the dose-volume histograms (DVHs) for primary gross tumor volume (PGTVnx) and planning target volume (PTVnx), similar to the intensity modulated radiation therapy (IMRT). The median follow-up duration was 43 months. Locoregional relapse and distant metastases as the first treatment failure events occurred in 32 (32/255, 12.5%) and 20 (30/255, 11.8%) patients, respectively. The 3-year and 5-year local control, disease-free survival, and overall survival rates were 83.3%, 82%, 83.8%, and 76.1%, 73.2%, 76.3% respectively. Univariate analysis displayed that clinical stage, T-stage, N-stage, and tumor response were related to prognosis. Multivariate analysis indicated that age, T-stage, N-stage, and combined chemotherapy were independent prognosticators. The incidence of grade 1–2 acute mucositis and leukocytopenia were 93.7% and 91.0%, respectively, with no cases of grade 4 toxicity detected.

**Materials and Methods:**

From November 2007 to December 2011, 255 patients with histologically diagnosed, non-metastatic NPC were enrolled into this study and received 4F-CRT. Magnetic resonance imaging scans of the nasopharynx were performed on every patient. All patients received definitive radiotherapy with 6 MV X-rays using conventional fractions at 2 Gy daily, 5 fractions per week, and 231 patients with stage IIb-IV received concurrent chemotherapy and cisplatin-based adjuvant chemotherapy. The accumulated survival was calculated according to the Kaplan-Meier method; the log-rank test was used to compare survival differences. Multivariate analysis was performed using Cox's proportional hazard model.

**Conclusions:**

Compared with the conventional treatment plans, the 4F-CRT plan delivered more dose to cover the tumor volume and reduces the doses of the normal tissues including the parotid gland, TMJs and so on. The long-term efficacy of 4F-CRT is satisfactory and its toxicities are tolerable.

## INTRODUCTION

Nasopharyngeal carcinoma (NPC) is one of the most common malignant tumors in Southern China [1, 2]. Owing to its anatomic location and high radiosensitivity, radiotherapy (RT) has long been as the preferred method of treatment for NPC. Conventional 2-dimensional radiotherapy (2D-RT) had been used as the most common method. Early literatures reported that the 5-year overall survival rates of stages I, II, III, and IV were 70–95%, 65–83%, 54–76%, and 29–56%, respectively [3–7]. With increasing in the number of survivors with NPC, more patients are experiencing various radiation related complications [8, 9], such as serious mouth dry, trismus, inner ear hearing loss, and brain injury, which decreased their quality of life.

Intensity modulated radiation therapy (IMRT), as a new approach, is preformed to the treatment planning and delivered the radiation for patients with cancer [10, 11]. Compared with 2D-RT and 3-dimensional radiotherapy (3D-CRT), IMRT improves the conformal dose covered the clinical target volume (CTV) in three dimensions, while protecting the normal tissues around the CTV. Recently published phase III studies indicated that IMRT has better curative effect and lower toxicity than with conventional 2D-RT or 3D-CRT [12, 13]. Therefore, IMRT is the first choice of radiotherapy for nasopharyngeal carcinoma because of its dosimetric advantages. However, there were more and more NPC patients in our hospital and only the current machines are not enough to meet all the NPC patients received IMRT. In order to overcome this difficulty, the author designed a method of the four facio-cervical fields conformal radiotherapy (4F-CRT) to treat patients with NPC [14] and applied this method in clinical practice according to the actual situation of the hospital. The purpose of our study was to analyze the long-term efficacy among 255 patients with NPC who received 4F-CRT in our hospital.

## RESULTS

### Plan comparison

**Figure 1:**
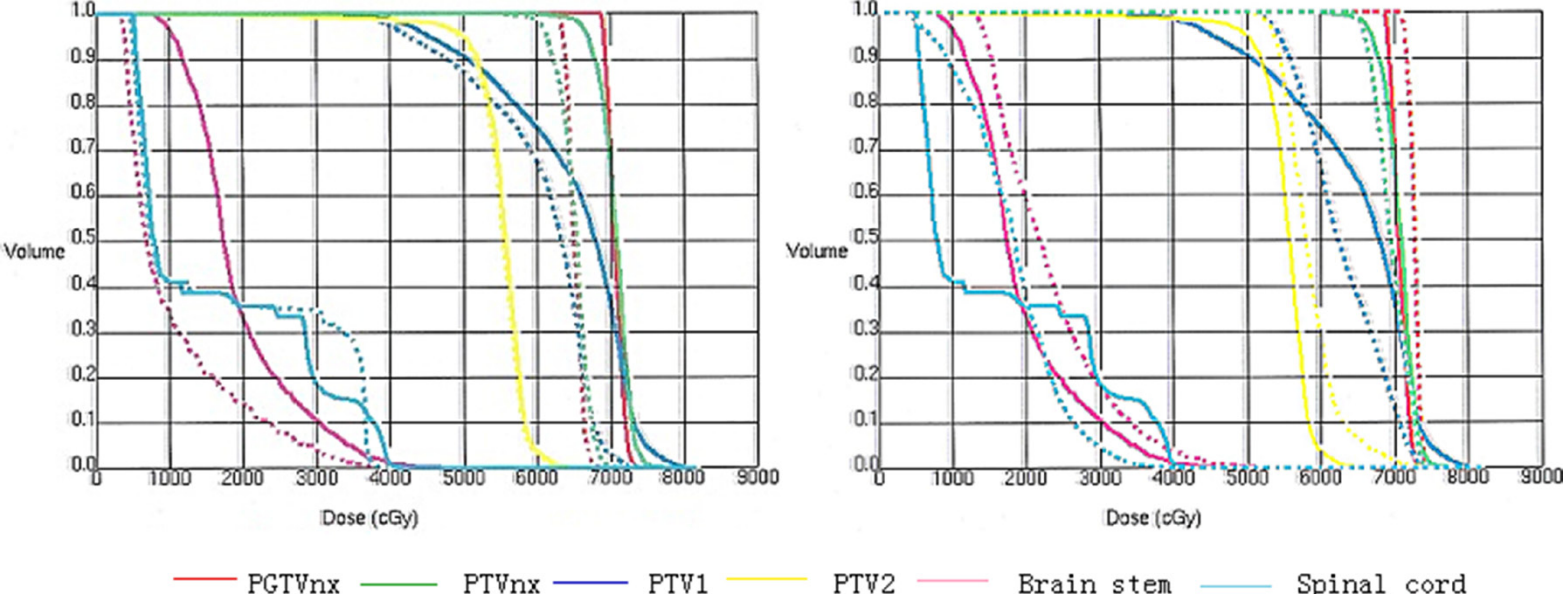
The DVHs of targets, brain stem, and spinal cord (Left: —4F-CRT vs. ...2D-RT; right: —4F-CRT vs. ...IMRT).

**Figure 2:**
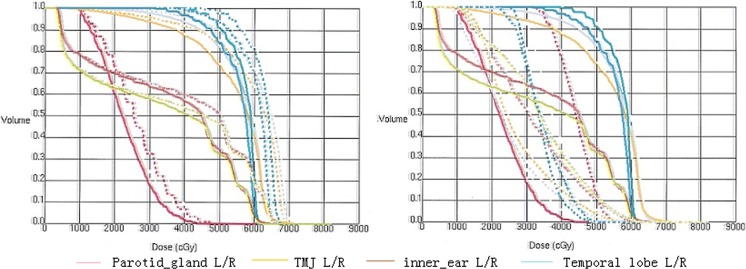
The DVHs for the parotid gland, temporomandibular joint (TMJ), inner ear, and temporal lobe (Left: —4F-CRT vs. ...2D-RT; right: —4F-CRT vs. ...IMRT).

One patient out of 255 underwent 3 different RT treatment modalities. The plans included 2D-RT, 4F-CRT, and IMRT. The DVHs of targets, brain stem, and spinal cord were shown in Figure 1. The sharper DVHs for primary gross tumor volume (PGTVnx) and planning target volume (PTVnx) of the 4F-CRT techniques were similar to those of the IMRT. Among the three plans, the mean doses of D_max_ received by spinal cord and brain stem were within safety limits. The DVHs of three plans for the parotid gland, temporomandibular joint (TMJ), inner ear, and temporal lobe were reported in Figure 2. Compared with the conventional 2D-RT plans, the 4F-CRT plan delivered lower dose to the OARs and protected parotid gland, TMJ, inner ear, and temporal lobe to avoid severe damage. However, there were more hotspot areas in the OARs for 4F-CRT than for IMRT.

### Response of tumor

The overall response rates for lesions of nasopharynx and cervical lymph nodes were 100% (complete remission [CR] 82.5%) and 100% (CR 95.7%) 3 months after completing RT, respectively.

### Treatment outcome and failure patterns

**Figure 3:**
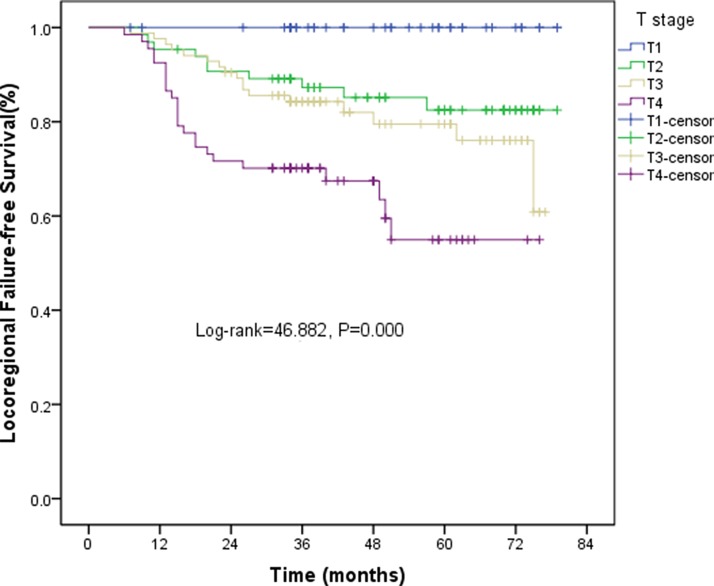
Kaplan-Meier curves of LRFS for T stage

**Figure 4:**
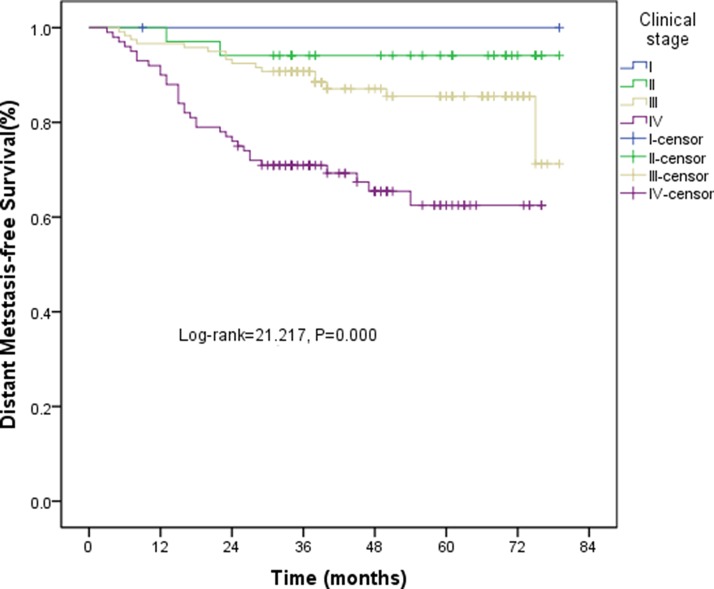
Kaplan-Meier curves of DMFS for clinical stage

All patients completed the 4F-CRT. The median follow-up was 43 (range, 8–79) months and 103 patients survived for more than five years. The three-year estimated local recurrence-free survival (LRFS), progression-free survival (PFS), and overall survival (OS) rates were 83.3%, 82%, and 82.8%, respectively. The five-year estimated LRFS, PFS, and OS rates were 73.2%, 76.3%, and 76.1%, respectively. The overall 5-year LRFS rates for the patients were 100%, 82.5%, 76%, and 54.9% for stage T1, T2, T3, and T4, respectively (log-rank = 46.882, *P* < 0.001) (Figure 3). The overall five-year distant metastasis–free survival (DMFS) rates for the patients were 100%, 94.1%, 85.5%, and 62.5% for stage I, II, III and IV, respectively (log-rank = 21.217, *P* < 0.001) (Figure 4).

Of all patients, 62 patients experienced treatment failure: 18 patients had local recurrence only; 10 developed regional relapse only and received salvage surgery; 4 had locoregional recurrence; 25 developed ≥ 1 distant metastasis, all of whom died from disease progression; and 5 patients occurred with locoregional relapse and distant metastasis. These details are shown in Table 1.

**Table 1:** Site and incidence of treatment failure

Sites	Number of patients (*n* = 62)
Local only	18
Regional only	10
Local and regional	4
Locoregional and distant	5
Distant only	
Lung only	5
Liver only	3
Bone only	4
Lung, bone, liver	13

### Risk factors analysis

Potential prognostic factors, including age, gender, histology, clinical stage, T classification, N classification, combined chemotherapy, blood group, weight loss, comorbidity, and tumor response, were evaluated using univariate analysis and Cox proportional hazards models. Univariate analysis revealed that T stage, N stage, tumor response, and clinical stage were significant predictors for overall survival (Table 2). However, age, T-classification, N-classification and combined chemotherapy were founded to be independent factors of prognosis for overall survival (Table 3).

**Table 2:** Impact of prognostic factors on treatment outcome by univariate analysis

Variable	*N*	5-year OS	χ^2^	*P*
Sex			1.801	0.18
Male	186	78%		
Female	69	85.5%		
Age (years)			6.314	0.12
≥ 60	66	69.7%		
< 60	189	83.6%		
Histology			2.434	0.487
WHO I	5	100%		
WHO II	244	79.1%		
WHO III	6	100%		
T stage			25.008	< 0.001
T1	39	100%		
T2	65	84.6%		
T3	84	79.8%		
T4	67	64.2%		
N stage			16.861	0.001
N0	18	83.3%		
N1	74	87.8%		
N2	117	82.1%		
N3	46	60.9%		
Clinical stage			22.622	< 0.001
I	2	100%		
II	34	94.1%		
III	119	86.6%		
IV	100	67%		
Weight loss (kg)			0.134	0.715
≥ 5	43	76.7%		
< 5	211	80.6%		
Combined chemo			0.608	0.434
Yes	231	80.9%		
No	24	72%		
Blood group			1.505	0.681
A	72	75%		
B	55	81.8%		
AB	27	85.2%		
O	101	81.2%		
Comorbidity			0.873	0.35
Yes	65	78.9%		
No	190	81.3%		
Tumor response			5.033	0.025
CR	174	83, 3%		
PR	81	72.8%		

**Table 3:** Influence of prognostic factors on treatment outcome by Cox proportional hazards models

Variable	B	SE	Wald	Odds ratio	*P*	95% CI
T stage	0.89	0.254	12.287	2.435	< 0.001	1.480–4.004
N stage	0.864	0.246	12.313	2.373	< 0.001	1.464–3.845
Age	0.04	0.014	8.291	1.041	0.004	1.013–1.069
Comb CT	0.918	0.446	4.228	0.379	0.04	0.167–0.958

Note: comb CT combined chemotherapy.

### Toxicity

Acute toxicities were assessed during treatment and late toxicities with 6 months follow-up (Table 4). No treatment-related death occurred. The most commonly observed acute toxicities included mucositis, skin reactions, xerostomia, leucocytopenia, and weight loss. The incidence of acute mucositis grade 1–2 was 93.7% (239/255), with no cases of grade 4 toxicity detected. No grade 3 or 4 skin reactions were occurred within the RT field. The incidence of grade 1–2 leucocytopenia was 91.0% (232/255). Five patients developed grade 4 leucocytopenia. However, the neutrophil count recovered rapidly following administration of granulocyte colony-stimulating factor (GCSF). No cases of renal function impairment were observed. Overall, 211 patients experienced mild to moderate weight loss.

**Table 4:** Frequencies of the most common acute and late treatment related toxicities by type and grade

Treatment toxicities	Total	Grade 1	Grade 2	Grade 3	Grade 4
Acute toxicities, n (%)					
Mucositis	250 (98.0)	50 (19.6)	189 (74.1)	11 (4.3)	0
leucocytopenia	240 (94.1)	137 (53.7)	95 (37.3)	3 (1.1)	5 (2.0)
Dematitis	245 (96.1)	214 (83.9)	23 (9.1)	8 (3.1)	0
Xerostomia	220 (886.3)	58 (22.7)	147 (57.7)	15 (5.9)	0
Late toxicities, n (%)					
Xerostomia *	165 (64.7)	72 (28.2)	82 (32.2)	11 (4.3)	0
Hearing loss	40 (15.7)	26 (10.2)	9 (3.5)	5 (2.0)	0
Neck fibrosis	95 (37.3)	89 (34.9)	6 (2.4)	0	0
REP	23 (9.0)	15 (5.9)	5 (2.0)	2 (0.7)	1 (0.4)

Note: REP Radiation encephalopathy; * The patients with NPC were evaluated at six months after radiotherapy.

Dry mouth was the most common late complication, and its severity decreased over time. The degree of xerostomia in most survivors was mild-to-moderate at the last follow-up time. 90 of all patients didn't subjectively complaint of xerostomia. 15.7% of patients developed unilateral or bilateral hearing impairment and 23 occurred radiation encephalopathy diagnosed by magnetic resonance imaging (MRI) at follow-up.

## DISCUSSION

Primary RT, as the mainstream of treatment, has been used to treat for patients with NPC. Ho [15] described that 2D techniques was used to deliver radical RT for NPC since the early 1990s. This was devised in the era when CT scans were not available and a shortage of machine time was a common problem in the community. Hence, all planning was done on simulator films using standard orthogonal field arrangements and lead blocks on the basis of anatomic landmarks rather than actual tumor geometry. The local control rates of NPC patients treated with 2D planning techniques were 80–85%, taking all T-stages together [16, 17]. With the emergence of the CT simulation and the 3D-TPS, the 3D relationship between the tumor and the surrounding OARs can be observed more vividly. It is obvious that this dose distribution of rectangular-shaped in 2D-RT planning is not ideal and will inevitably lead to undesirable target coverage and the coverage of a large number of normal tissues. A dosimetric study by Chau et al. [18] showed that the conventional 2D planning technique was inadequate in achieving dose coverage in the superior (skull base), posterolateral (parapharyngeal), and inferior regions of the target area, and the high dose areas of OARs. Ng et al. [19], who reviewed 700 patients with NPC, concluded that if target coverage could be improved through the use of a better RT technique, parapharyngeal extension would no longer be a significant prognosticator. Moreover, a quality of life survey showed that xerostomia, dysaudia, dysphagia, and trismus were regarded as the most frequent complications of disease-free NPC survivors treated with conventional 2D-RT techniques [20]. Hence, NPC patients delivered conventional 2D-RT techniques developed serious side effects and experienced very poor quality of life.

With the development of technology, 3D-CRT or IMRT with inverse RT planning should be as the modern RT technique used for NPC. Until the mid-1990s, IMRT has been used for the treatment of different head and neck carcinoma, including NPCs. IMRT techniques can provide better dose distributions for irregular- or concave-shaped target volumes near the OARs. Non-randomized studies demonstrated that compared with 2D-RT or 3D-CRT plans, IMRT provided the better tumor coverage and the lower dose of OARs including the central nervous system and parotid glands in the treatment of NPC [21–24]. Single institutional results showed that locoregional control and OS rates of NPC were enhanced by IMRT [25–29]. Despite as a new technology, it is encouraging to improve the therapeutic ratio, whether marginal relapse may be caused by the conformation of target coverage and the protection of normal tissue is not determined [30–32]. Though IMRT is the first choice to treat NPC patients, all patients cannot be used for IMRT owning to the lesser machines and more patients. So we designed an alternative therapeutic method to conventional 2D-RT and applied this method in clinical practice according to the actual situation of our hospital.

The dosimetric study showed 4F-CRT technique provided better tumor coverage and OARs sparing [14]. DVH showed that the sharper curves for PGTVnx and PTVnx in the 4F-CRT plans were similar to those in the IMRT. The doses of the target volumes in the 4F-CRT plans were higher than those in the 2D-RT plans. At the same time, the Dmax doses of OARs were within safety limits.

In total, 255 patients accepted the 4F-CRT plans and 231 received concomitant chemotherapy. The 3-year LRFS, PFS, and OS rates were 83.3%, 82%, and 82.8%, respectively. The 5-year LRFS, PFS, and OS rates were 76.1%, 73.2%, and 76.3%, respectively. Univariate analysis indicated that T stage, N stage, tumor response, and clinical stage were significant predictors for OS. However, age, T-classification, N-classification and combined chemotherapy were founded to be independent factors of prognosis for OS. The predominant acute toxicities were mucositis, skin reactions, xerostomia, leucocytopenia, and weight loss; most were mild or moderate. Hence, the long-term efficacy of 4F-CRT is satisfactory and its acute toxicities are tolerable. The results demonstrated that better clinical outcome and quality of life for NPC patients were obtained 4F-CRT due to the dosimetric advantage.

To the best of our knowledge, the present study is the first designed to test the outcome and long-term efficacy of 4F-CRT in patients with NPC. This regimen achieved good treatment results and demonstrated an acceptable acute toxicity profile. Furthermore, late complications (including severe xerostomia, hearing impairment, and temporal lobe necrosis) were less frequently observed in patients undergoing 4F-CRT than in those undergoing conventional 2D-RT. However, our findings are limited by virtue of being from a phase II clinical trial, restricted to China. The sample size was small.

## MATERIALS AND METHODS

### Patients and pretreatment evaluation

From November 2007 to December 2011, a total of 255 consecutive patients with newly histologically diagnosed, non-metastatic NPC received prospectively treatment with 4F-CRT at the Department of Radiation Oncology, Zhejiang Cancer Hospital (Hangzhou, People's Republic of China). The patients were aged 21–81 years, and 186 men and 69women were included. All patients underwent pretreatment evaluation which included a complete history, physical examination, hematology and biochemistry profiles, chest radiography, abdominal sonography, bone scan, and magnetic resonance imaging of the nasopharynx. All patients were staged according to the 2010 AJCC staging system [33]. Five patients had type I, 244 had type II, and 6 had type III tumors, according to the World Health Organization's classification of tumor histology [34]. The characteristics of the patients are summarized in Table 5.

**Table 5:** The clinicopathological characteristic of the 255 patients enrolled in the study

Patient characteristic	Number of patients (%)
Sex	
Male	186 (72.9%)
Female	69 (27.1%)
Age (years)	
Median (range)	52
≥ 60	66 (25.9%)
< 60	189 (74.1%)
WHO histologic type	
II	5 (2%)
III	244 (95.7%)
Other	6 (2.3%)
T stage	
T1	39 (15.3%)
T2	65 (25.5%)
T3	84 (32.9%)
T4	67 (26.3%)
N stage	
N0	18 (7.1%)
N1	74 (29%)
N2	117 (45.9%)
N3	46 (18%)
UICC stage	
I	2 (7.8%)
II	34 (13.3%)
III	119 (46.7%)
IV	10039.2%)
Combined chemotherapy	
Yes	231 (90.6%)
No	24 (9.4%)

### Delineation for the tumor volume

All patients were immobilized in the supine position with thermoplastic masks. Computed tomography scans with intravenous contrast, using 2.5 mm slices from the head to a level 2 cm below the sternoclavicular joints were performed for planning. Target volumes were delineated according to the recommendations by the International Commission on Radiation Units and Measurements (ICRU) CTV delineation protocol for head and neck malignancies [35, 36].

Gross tumor volume (GTV) referred to the tumor extent found in clinical and imaging examinations. The primary tumor extent including the metastatic retropharyngeal lymph nodes (RLNs) was named as GTVnx, and the metastatic lymph nodes of the neck as GTVnd.

Clinical target volume (CTV) was defined individually according to GTV, and risk region potentially involved around the nasopharyngeal cavity. The CTV for GTVnx included CTVnx for the high-risk clinical target volume and CTV1 for potentially invaded extension. CTVnx was defined as GTVnx plus a 7 mm margin, encompassing the entire nasopharyngeal mucosa plus 5 mm submucosal volume. For CTV1, potentially involved anatomic regions, including the whole nasopharyngeal cavity, the anterior one- to two-thirds of the clivus (when invaded, the whole clivus should be covered), the skull base, the pterygoid plates, the parapharyngeal space, the inferior sphenoid sinus (the whole sphenoid sinus should be covered for stages T3 and T4), the posterior one-quarter to one-third of the nasal cavity, and the maxillary sinus, were included. Level Ib was considered high risk in patients with metastatic lymph nodes in level IIa, and any lymph node drainage pathways containing metastatic lymph nodes were considered high risk. Prophylactic low risk neck irradiation areas were referred as CTV2, including levels IV and Vb without metastatic cervical lymph nodes.

**Figure 5:**
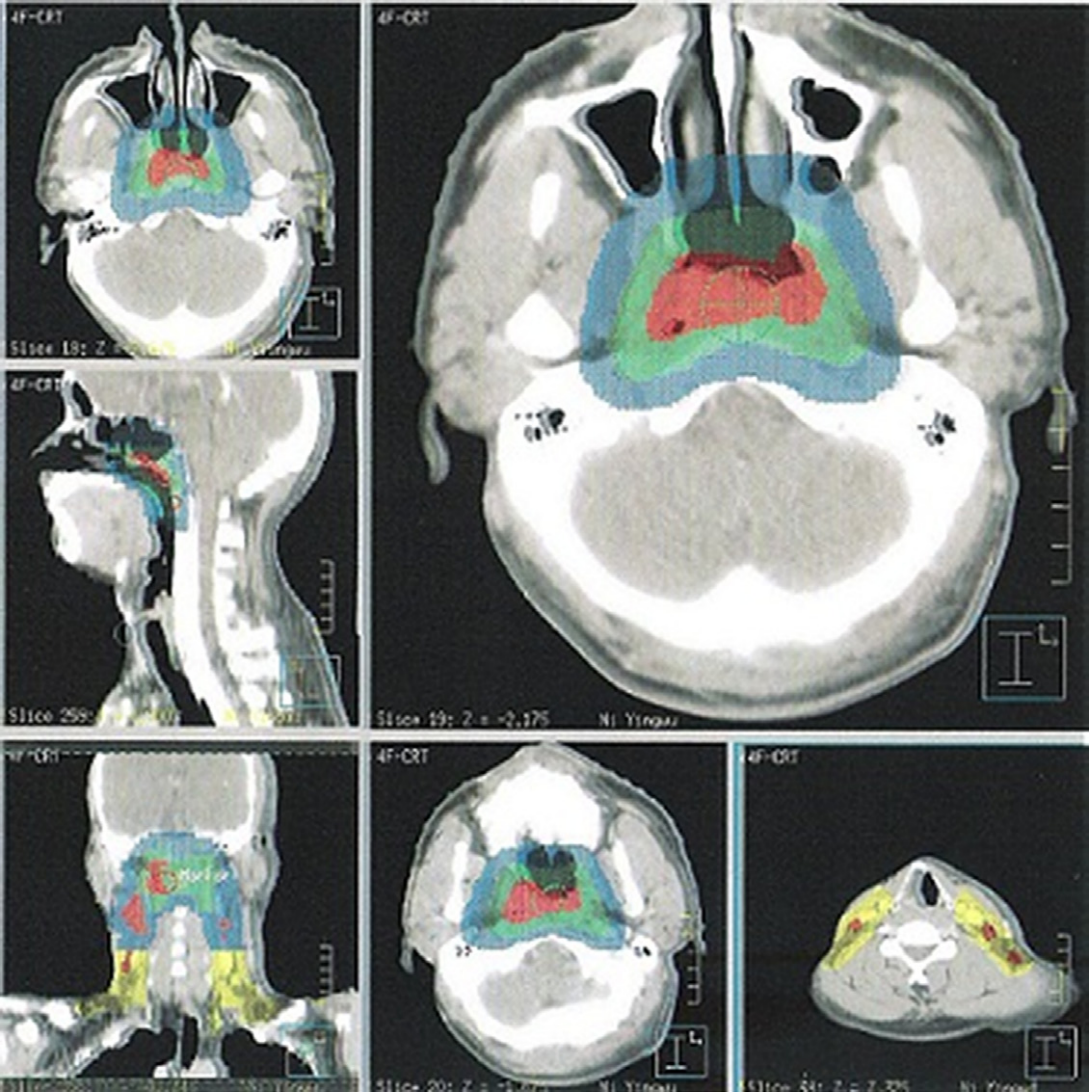
Selected CT slides for demonstrating the delineation of target volumes (Red area: PGTVnx and GTVnd; Green area: PTVnx; Blue area: PTV1; Yellow area: PTV2).

The planning target volume (PTV) was constructed automatically based on each volume with an additional 3-mm margin in three dimensions, allowing for setup variability. All of the PTVs including PGTVnx, PTVnx, PTV1 and PTV2, should not be delineated outside of the skin surface. Figure 5 demonstrates the delineations of target volumes.

Critical normal structures, including the brainstem, spinal cord, parotid glands, optic nerves, chiasm, lens, eyeballs, temporal lobes, temporomandibular joints, mandible, and hypophysis were contoured and set as OARs during optimization.

### Radiotherapy techniques

Treatment, given in three phases, was performed as formerly described [14]. Four facio-cervical (anterior and posterior facio-cervical and two lateral opposing facio-cervical) fields were irradiated for CTV1 with 34 Gy/17 F and a lower cervical anterior tangent field was used for CTV2 with 34 Gy/17 F for phase I treatment. The eyes, optic nerves, temporal lobe, and brain stem were shielded as much as possible. For phase II treatment, treatment could be continued using the two lateral opposing facio-cervical fields but with shrinkage of fields to avoid the spinal cord and superior neck, and superior-lower neck lymphatics with electron fields. A dose of 26 Gy was given to CTV1, while a dose of 20 Gy was used for CTV2. For phase III treatment, bilateral preauricular fields covered CTVnx with a dose of 10 Gy. The eyes, optic nerves, and brain stem were shielded.

### Chemotherapy

Overall, 231 patients were eligible for chemotherapy as they met the following criteria: ECOG performance status ≤ 2, white blood cell count ≥4000 cells/μL, and platelet count > 10,000/μL. In concurrent CRT, two courses of chemotherapy were planned, consisting of cisplatin (30 mg/m^2^/day) and 5-Fu (500 mg/m^2^/day) for 3 days. In the following adjuvant chemotherapy, two cycles of chemotherapy were planned, consisting of cisplatin and 5-Fu.

### Plan evaluation

Plans were compared by target coverage according to the cross-section dose distribution and the dose-volume histogram (DVH) of targets. Parameters of the DVH were evaluated as following: 1) the doses received by the 95% and 90% volumes of PTV (D95 and D90), maximum PTV dose (Dmax), minimum PTV dose (Dmin), mean PTV dose; 2) maximum OARs dose and the volume of OARs received high dose. Moreover, the time was measured between the patient entering and exiting the treatment room for completing each of three plans. This included time for patient to change, set-up time, EPID time (once weekly), and the actual treatment time.

### Toxicity and survival evaluation

Toxicity was evaluated using the common toxicity criteria of the National Cancer Institute. Survival was calculated from the date of diagnosis to the date of most recent follow-up, recurrence, or death. The pattern of failure was defined according to the first site of failure: local failure was defined as recurrence of the primary tumor or metastasis to regional lymph nodes; and distant failure indicated metastasis to any site beyond the primary tumor and regional lymph nodes.

### Statistical analysis

Survival curves were performed using Kaplan-Meier product-limit methods. Comparison of the curves was performed using the log-rank test. Multivariate analysis to identify significant prognostic factors was accomplished using Cox regression models. Hazard ratios (HR) and 95% confidence intervals (CI) were calculated for each prognostic factor to identify those with statistical significance. IBM SPSS statistics version 19.0 software was used for all data analysis. Statistical significance was indicated at P <0.05. Survival time was calculated from the date of diagnosis to the most recent follow-up or to the date of relapse (event-free, local recurrence-free, or distant metastasis-free) or death (overall survival). After recurrence or metastasis, patients were given salvage therapy as determined by their physicians.

## CONCLUSIONS

This study on 4F-CRT used for the treatment of NPC had indicated that the superiority of 4F-CRT in achieving tumor coverage, dose homogeneity, and sparing of normal organ. In our hospital, this technique had been accepted as a treatment method to replace conventional 2D-RT in the treatment of NPC. The major superiority of 4F-CRT is to reduce the radiation dose of several OARs. Moreover, this regimen demonstrates better long-term efficacy and an acceptable acute toxicity profile. This study was a single center trial and has all the limitations of single center.
